# Lithium Therapeutic Functions: An Update on Pharmacokinetics, Pathophysiological Mechanisms of Action, Toxicity, and Side Effects

**DOI:** 10.1007/s12035-026-05663-9

**Published:** 2026-02-02

**Authors:** Amanda Gollo Bertollo, Maiqueli Eduarda Dama Mingoti, Sales Antonio Barbosa Junior, Paula Dallagnol, Paula Teresinha Tonin, Zuleide Maria Ignácio

**Affiliations:** 1 Graduate Program in Neuroscience, Federal University of Santa Catarina (UFSC), Florianópolis, SC Brazil; 2 Laboratory of Physiology, Pharmacology, and Psychopathology, Graduate Program in Biomedical Sciences, Federal University of Fronteira Sul (UFSC), Chapecó, SC Brazil; 3 University of Western São Paulo (UNOESTE), Presidente Prudente, SP Brazil; 4 Ingá University Center (UNINGÁ), Maringá, PR Brazil

**Keywords:** Lithium, Bipolar disorder, Mood regulator

## Abstract

Lithium is the most commonly used medicine to treat bipolar disorder (BD). It is considered a mood regulator, and the mechanisms underlying this effect still need to be elucidated. Some modulations are involved in neuroprotection, including neuronal communication, neuron differentiation and survival, synaptic modulation and plasticity, modulation of cognition, contribution to antioxidant defense, and reduction of inflammation, glial dysfunction, and apoptosis. In general, about 50% of the concentrations in serum are in the brain. However, it is essential to note that many gene expression differences influence the concentrations and actions of individuals. This review discusses the various mechanisms of lithium in BD I and II, its effects on neurotransmitters and receptors, the hypothalamic–pituitary–adrenal (HPA) axis, inflammation and neuroinflammation, immune functions, oxidative and nitrosative stress, mitochondrial respiratory chain function, intracellular signaling, and brain plasticity, as well as toxicity and side effects.

## Introduction

Lithium is a chemical element with the symbol Li, atomic number 3, and atomic mass 7. Its name derives from the Greek (lithos), which means stone. In nature, it never appears freely [64]. The biological significance of lithium is based on the use of salts for the treatment of psychiatric disorders. Lithium has been an important therapeutic option, especially for mood disorders, since its first clinical study by MogensSchou in 1954 [96]. After nearly 70 years of research, lithium salts are effective in protecting against mania and depression, especially in the acute and long-term management of bipolar disorder (BD) [41].

It is a drug classified as a mood modulator. Its use decreases symptoms of mania and depression, controls mood, and reduces the risk of suicide [72]. Its mechanism of action still needs to be fully elucidated. Studies indicate that lithium inhibits the expression and activity of the sodium/myo-inositol transporter (SMIT) system, thus limiting the entry of inositol into the cell. This substance acts as a facilitator in neuronal communication. Consequently, lithium ingestion culminates in alterations of several inositol-related pathways and targets [72]. In addition, there is modulation of signal transduction of the signaling pathways of glycogen synthase kinase 3 (GSK-3), protein kinase C (PKC), mammalian target of rapamycin (mTOR), wingless-related integration site (Wnt), erythroblastic leukemia viral oncogene homolog (ErbB), mitogen-activated protein kinase (MAPK), and vascular endothelial growth factor (VEGF) [1].

Lithium stimulates neurotrophic cell cascades, which are responsible for mediating neuron differentiation and survival, as well as synaptic modulation and plasticity. These functions are mediated by several substances, including brain-derived neurotrophic factor (BDNF), nerve growth factor (NGF), and B-cell lymphoma 2 (Bcl-2). A GSK3 is involved in the modulation of important intracellular signaling pathways, among them the pathway of protein kinase B (PKB/Akt), which is responsible for regulating cell survival, and the Wnt signaling pathway, which is involved in numerous cellular processes, including apoptosis and cell proliferation [22]. In addition, this enzyme plays an important role in the inflammatory response, given its essential action in stimulating certain pro-inflammatory cytokines, such as interleukin-1β (IL-1β), IL-6, and tumor necrosis factor alpha (TNF-α), as well as in decreasing the anti-inflammatory cytokine IL-10 [15]. Therefore, considering that lithium inhibits GSK3, this compound may be acting to reduce the inflammatory process. Furthermore, a good clinical response to lithium has been positively correlated with the normalization of the immune system [110].

Another action of this drug lies in its immunoregulatory effects on various receptors and substances, such as the B cell receptor, T cell receptor, and chemokine signaling pathways [1]. An in vitro study identified, in lipopolysaccharide (LPS)-activated Raw 264.7 macrophages, that lithium modulates the expression of several inflammatory genes that regulate the nuclear factor Kappa B (NF-κB) pathway, in addition to reducing the action of NF-κB by decreasing cell translocation [71].

The literature suggests that the pathways facilitated by lithium modulate energy metabolism, providing neuroprotective actions and promoting neuroplasticity. The primary activities involve modulating transcription factors, regulating specific gene expression, and controlling cytoskeletal function, as well as balancing neurotransmitter levels and adjusting fluctuations in second messengers and protein kinases. These and other functions are developed to guarantee the drug’s therapeutic effect [58]. Despite these established actions, key unresolved questions persist, including: (1) the precise reasons for variable patient response, (2) the mechanism by which lithium's multiplicity of targets converges on a specific therapeutic outcome, and (3) the physiological boundary between lithium's neuroprotective and potential neurotoxic effects, which are critical gaps addressed throughout this review.

## Main Therapeutic Functions of Lithium in the BD

Lithium is the primary recommended medication for the treatment of BD I [2, 44]. However, its specific action in mood regulation is not yet fully understood [118]. Despite its widespread use, there is still no biological marker that can specifically demonstrate its effects, highlighting the importance of understanding its mechanisms of action in the body [2]. Understanding these mechanisms is fundamental not only to enhance therapeutic outcomes but also to develop more targeted treatments for BD.

Altered oxidative stress levels have been documented in the post-mortem prefrontal cortex, as well as in the peripheral blood cells of patients diagnosed with BD [5, 48]. Among the multiple factors contributing to the development of BD, the physiological imbalance involving oxidative stress is considered a determining process [70]. Lithium, as a mood-stabilizing compound, exhibits the ability to confer protection against oxidative stress [39]. This antioxidant effect may play an important role in preventing cellular damage and promoting long-term brain health.

At the cellular level, lithium’s mood-stabilizing effects result in neuroprotection through its actions on neuroprotective pathways [118]. It also regulates BDNF, thereby preventing cellular degeneration [36]. Lithium is known to act on the mitochondria by reducing inosine levels and inhibiting inosine monophosphate 1. These effects promote increased neuroplasticity and modulate the neurotransmitters glutamate, dopamine, gamma-aminobutyric acid (GABA), acetylcholine, and glycine [118]. Such broad neuromodulatory activity contributes to lithium’s efficacy in stabilizing mood and reducing the recurrence of affective episodes.

Within the glutamatergic pathway, lithium acts on both presynaptic terminals and postsynaptic currents, exerting an inhibitory effect [113]. It also exerts neuroprotective effects by directly or indirectly inhibiting the gene expression of glycogen synthase kinase 3β (GSK3) [56, 57]. The indirect inhibition of GSK3 activity occurs through the activation of the AKT kinase family, resulting in inhibitory phosphorylation at the N-terminal region and suppression of phosphatases responsible for GSK3 dephosphorylation [38]. This mechanism has been associated with improvements in depressive symptoms [32]. Consequently, there is a reduction in cellular apoptosis due to the inactivation of GSK3β [12]. Given the centrality of GSK3 in various signaling pathways, its modulation by lithium may represent a key target for understanding mood stabilization.

Direct inhibition occurs through competition between Li⁺ and Mg^2^⁺ ions, which prevents Mg^2^⁺ from binding to GSK3 kinases, where it normally functions as a cofactor [106]. Studies analyzing peripheral blood from BD patients treated with lithium have shown increased levels of inhibitory phosphorylation of GSK3 at the N-terminal, which is associated with the reversal of depressive symptoms [50]. Nevertheless, this complex mechanism cannot be simply explained, as multiple cellular processes are involved in GSK3 activity [24]. Therefore, lithium’s effect on GSK3 is likely to be part of a broader network of biochemical interactions contributing to its therapeutic action.

A study evaluating BD I patients without acute episodes, but with an indication for long-term treatment, found lithium to be more effective in preventing acute episodes [42]. In this regard, lithium has also proven effective in preventing the recurrence of manic episodes [118]. These clinical findings underscore lithium’s preventive efficacy, which remains one of its most valuable features in long-term management.

In a longitudinal study involving 369 patients with BD I and BD II over one year, lithium was shown to be more effective in the treatment of BD II [107]. Another study assessing gray matter volume in BD patients inferred improved neuroplasticity and resilience, along with an association between continuous lithium use and increased gray matter volume [12]. Such structural brain changes further support the neurotrophic hypothesis of lithium’s action.

Supporting this, a study using mice examined the types of brain cells involved in cell proliferation after lithium treatment using stereological methods. The results indicated an increased number of neurons and glial cells in the dentate gyrus, along with higher astrocyte density [91]. This evidence of enhanced neurogenesis reinforces the potential regenerative effects of lithium at the cellular level.

More recent studies have focused on the role of the ankyrin 3 (ANK3) gene, which encodes the adaptor protein Ankyrin-G (AnkG). This gene has been linked to the pathophysiology of BD, especially through its involvement in neurotransmission and neuroplasticity regulation pathways. Lithium has demonstrated the ability to reverse behavioral disturbances resulting from impaired AnkG functionality [50].

In a study evaluating the kynurenine pathway, lithium exhibited anti-inflammatory effects by reducing the activity of indoleamine 2,3-dioxygenase (IDO1), a process regulated by the GSK3 inhibitor SB-216763 in primary human microglial cells and hiPSC-derived microglia [45]. This immunomodulatory action may contribute to its efficacy in stabilizing mood by reducing inflammation-related neurotoxicity.

Finally, pharmacogenetics has been increasingly used to enhance the precision of lithium therapy, offering a perspective that helps predict whether a patient will respond favorably to the medication [2].

### Translational Insights from Clinical Trials

#### Lithium Efficacy in Bipolar Disorder Type I Versus Type II

Lithium is recognized as a first-line therapy in the management of BD, especially type I [46]. In this sense, clinical trials were sought to evaluate the efficacy of lithium in BD type I versus type II, revealing a need for more research to elucidate this relationship. Considering the above, it was not possible to identify clinical trials that clarified the effective use of lithium in BD I and BD II, nor trials that specifically evaluated or differentiated efficacy and doses between the acute and maintenance phases.

#### Neuroimaging Correlates

When evaluating the use of lithium and correlations with changes in brain structure, a study evaluating BD I patients through MRI images identified an increase in the volume of the amygdala, thalamus, and hippocampus in lithium-treated patients compared to unmedicated individuals and healthy controls, hypothesizing that this result may be related to neuroprotection and neurotrophic mechanisms. Lithium may induce regenerative processes in specific brain regions, particularly the hippocampus. These findings may be indirectly associated with evidence from studies demonstrating elevated BDNF levels in patients taking lithium [65].

However, Sarrazin et al. [95] tested the hypothesis that dendritic density is higher in patients on lithium therapy than in those without lithium, using advanced MRI-based modeling of water diffusion in 41 medicated patients and 40 controls. The results revealed a significant group effect in the left prefrontal region: patients without lithium showed lower frontal neurite density than controls, whereas those on lithium showed higher mean neurite density than non-users. This finding suggests that variations in the intracellular volume fraction reflect microstructural reorganization of gray matter, supporting the hypothesis that lithium exerts a beneficial effect on the human neuronal compartment.

#### Biomarkers Associated with Response

In evaluating biomarkers associated with lithium use, some findings are noteworthy, though further elucidation through clinical studies of this relationship with biomarkers and cellular responses is needed. A survey in BD I/II patients under lithium treatment (16 weeks) utilized CellPrint flow cytometry to quantify levels of multiple intracellular proteins in CD4 + lymphocytes and monocytes, comparing responders and non-responders in monotherapy for BD I or II to explore potential predictive biomarkers of therapeutic response. CellPrint flow cytometry revealed that low levels of phosphorylated nuclear factor NF-kappa-B p65 subunit (phospho-RelA) and GSK3β may be involved in metabolic pathways and could improve the sensitivity and specificity of predicting lithium response. It was also observed that phospho-RelA and GSK3β are implicated in signaling pathways for prolactin, leptin, BDNF, and neurotrophins [40]. Furthermore, no robust evidence was found that GSK-3β phosphorylation, BDNF levels, or inflammatory markers predict therapeutic response between individuals with bipolar disorder type I and type II [73, 40].

#### Trials with Low-Dose Versus Standard-Dose Lithium

Considering the dose of lithium, essential findings were raised in a clinical trial, noting that lithium side effects are often challenging for clinical management. Forlenza et al. [37], in a double-blind, placebo-controlled clinical trial of low-dose lithium (0.25–0.5 mmol/l) in patients with mild cognitive impairment, aimed to delay cognitive decline and prevent progression to dementia. Beneficial effects on cognitive function and biomarkers were observed in individuals with amnestic mild cognitive impairment and Alzheimer’s disease. Therefore, studies suggest that the use of low-dose lithium may contribute to reducing progression to dementia in individuals with mild cognitive impairment without the burden of significant side effects [8].

## Pharmacokinetic Profile of Lithium

**Fig. 1:**
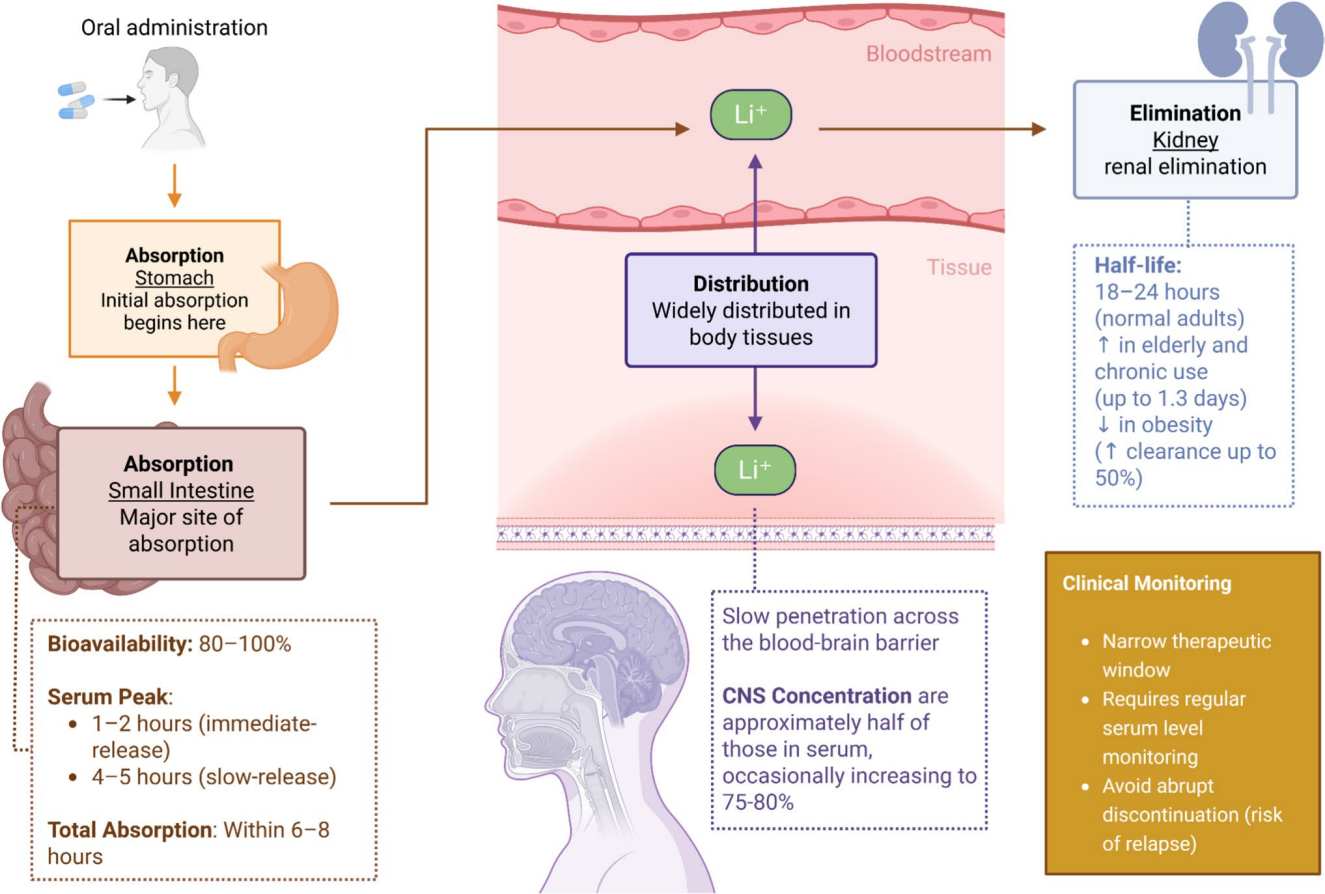
Pharmacokinetic profile of lithium. After oral administration, lithium is rapidly and completely absorbed in the gastrointestinal tract, with a bioavailability ranging from 80% to 100%. Serum peak is reached within 1–2 h (immediate-release) or 4–5 h (slow-release). Total absorption occurs within 6–8 h. Lithium does not bind to plasma proteins and undergoes no metabolic transformation. It is widely distributed throughout body tissues, including the central nervous system (CNS), where penetration is slow. CNS concentrations are approximately 50% of serum levels, occasionally reaching 75%–80%. Elimination is primarily renal, with a half-life of 18–24 h in healthy adults, which may be prolonged in the elderly or during chronic use (up to 1.3 days) and reduced in obesity (with clearance increased by up to 50%). Due to its narrow therapeutic window, lithium treatment requires regular serum level monitoring and gradual discontinuation to reduce the risk of relapse. Created in BioRender

Lithium has a narrow therapeutic window and is wholly and rapidly absorbed orally, with bioavailability ranging from 80 to 100% (Fig. 1). It reaches serum peaks between 1 and 2 h in the usual preparations and between 4 and 5 h in those of slow and controlled release. It is completely absorbed in 6 to 8 h. It has no binding properties to plasma proteins or metabolites [47].

The kidneys mainly carry out excretion, although small amounts are expelled through feces and sweat. The elimination half-life is 18 to 24 h; however, in the elderly, it can be prolonged due to decreased glomerular filtration. In addition, continuous use for more than one year increases the half-life of the drug to 1.3 days, and on the other hand, obesity can decrease the half-life, increasing clearance by up to 50% [47].

Lithium is widely distributed in all tissues, but to a variable extent. The entry and exit of lithium in the central nervous system (CNS) are slow, and brain concentrations are approximately half of those in serum, occasionally increasing to 75–80%. This may be why some acute overdoses are relatively well tolerated, and chronic intoxications persist even after a substantial decrease in serum levels [47].

When using this drug, laboratory control is essential both before and after treatment, considering that the therapeutic window for lithium is small. In addition, abrupt discontinuation of lithium increases the risk of relapse, so lithium must be withdrawn gradually if necessary. Lithium is generally safe, only contraindicated in a few specific conditions [9].

A survey of mother–infant pairs identified that lithium monotherapy might be a treatment option for pregnant women with BD if it is associated with binomial monitoring. The analysis involved nine mother–infant pairs exposed to the drug during late pregnancy and exclusive breastfeeding in the Perinatal Psychiatric Unit. It was verified that in pregnant women, there was no intoxication, and in the babies, there was no growth or development delay in the follow-up period, which was six months of the child's life [54].

## Pharmacogenetics of Lithium

Genetic variation can interfere with drug metabolism and is associated with varying therapeutic responses and adverse events [94]. Pharmacogenetics aims to identify genetic variants associated with the effectiveness of drugs. Studies have determined that other variants are associated with varying responses to lithium in individuals with BD [84].

The FKBP5 gene encodes proteins that increase sensitivity to glucocorticoid receptors. One study identified that the presence of the T allele in the rs1360780 variant of the FKBP5 gene in depressed individuals treated with lithium is associated with an increased risk of suicidal ideation compared to the C allele. The risk of suicidal ideation was correlated with the severity of depression in people with depression receiving lithium compared to individuals with the T allele in the rs130058 variant of the HTR1B gene, responsible for encoding the 5-hydroxytryptamine 1B receptor, which therefore plays an essential role in the serotonergic system [88, 119].

Polymorphisms in the gene encoding neurotrophic tyrosine kinase receptor type 2 (NTRK2) influence the effectiveness of the response to lithium. NTRK2 is a specific receptor for BDNF, modulating neuronal differentiation and cell survival. Regarding the rs2769605 variant identified in 27% of the sample, the CC genotype was associated with a decreased response to lithium in individuals with BD I compared to the CT + TT genotypes. The same research identified that the AA genotype of the rs1565445 variant of the same gene was present in 35% of the sample and was not associated with the response to lithium in people with BD I compared to controls [115].

Research in animal models indicates that lithium inhibits the brain activity of GSK-3 and the signal transduction it produces. In the GSK3B gene, the AA genotype of the rs334558 variant is associated with a decreased response of individuals with BD I treated with lithium for at least 24 months compared to the AG + GG genotypes. The survey was conducted with 138 subjects with a confirmed diagnosis and 131 controls [63]. Furthermore, the rs6438552 variant of the GSK3B gene with the GG genotype is associated with an increased response to lithium in people with BD, as is the rs2071427 variant of the NR1D1/THRA gene with the T allele compared to the C allele [76].

A clinical trial analyzed nucleotide polymorphisms in 2563 individuals with BD who had been using lithium as monotherapy for at least six months. The researchers identified that the T allele was associated with an increased response to lithium in people with BD compared to the C allele in the rs78015114 and rs79663003 variants, just as the G allele increased the drug response over the A allele in the rs74795342 variant [53].

The rs16909440 variant of the OR52E2 gene was analyzed in 52 patients with BD treated with lithium. It was found that the TT genotype is associated with a higher drug response compared to the CC genotype. Furthermore, regarding the rs16973410 variant, the CC genotype increased the response to lithium compared to the CT + TT genotype, and the GG genotype of the rs2499984 variant of the OR52J3 gene had the same effect as the AA genotype. Finally, in the rs11869731 variant of the ASIC2 gene, the CC genotype also increased the impact of lithium compared to the CG and GG genotypes [101].

A survey analyzed 2586 individuals with BD and identified that variants rs7588746, rs3919583, rs1611255, rs209474, rs1521470 of the ADCY1 gene, rs79403677 of the FAM177A1 gene, rs7959663 of the MYO1H gene, rs66486766, rs6728642 of the FAM178B gene, rs324899, rs6942227, rs1611259, and rs61123830 of the GRAMD1B gene were decreasing the response to lithium. The variants rs7405404 and rs62200793 of the gene ZNF804A and rs59724122 increased the response to lithium [3].

Considering that pharmacogenetics interferes with the response to lithium, it is essential to consider customizing psychopharmacological therapy for patients. Research with larger samples and in different regions is needed to elucidate the genes and polymorphisms related to the action of lithium globally, and thus, to be able to implement the pharmacogenetic evaluation of individuals in clinical practice, ensuring that lithium has the necessary efficacy.

Genome-wide association studies (GWAS) and polygenic risk scores (PGS) have consistently shown that genetic factors contribute to the variability in lithium response among patients with bipolar disorder. Pathway-specific PGSs, targeting acetylcholine, GABA, calcium channel, mitochondria, circadian rhythm, and GSK pathways, are associated with lithium response, but each explains only a small fraction (0.29%–1.91%) of the variance. Combining these scores increases the explained variance to about 3.7% for categorical outcomes, which is comparable to conventional genome-wide PGSs but with better biological interpretability. Notably, patients with the highest genetic loading for acetylcholine pathway variants are about three times more likely to respond well to lithium than those with the lowest loading [4, 99].

Despite these advances, single-nucleotide polymorphisms (SNPs) and PGSs alone have low predictive power for individual patients, and findings often lack replication across diverse cohorts [53, 89, 97, 111]. The clinical utility of these genetic markers remains limited, as current models do not achieve the accuracy needed for routine clinical use [85, 86, 89]. Integrating genetic data with robust clinical variables and machine learning approaches can improve prediction, with some models explaining up to 13.7% of the variance in lithium response, but these require further validation [4, 23, 82].

## Lithium, Neurotransmitters, and Receptors

**Fig. 2:**
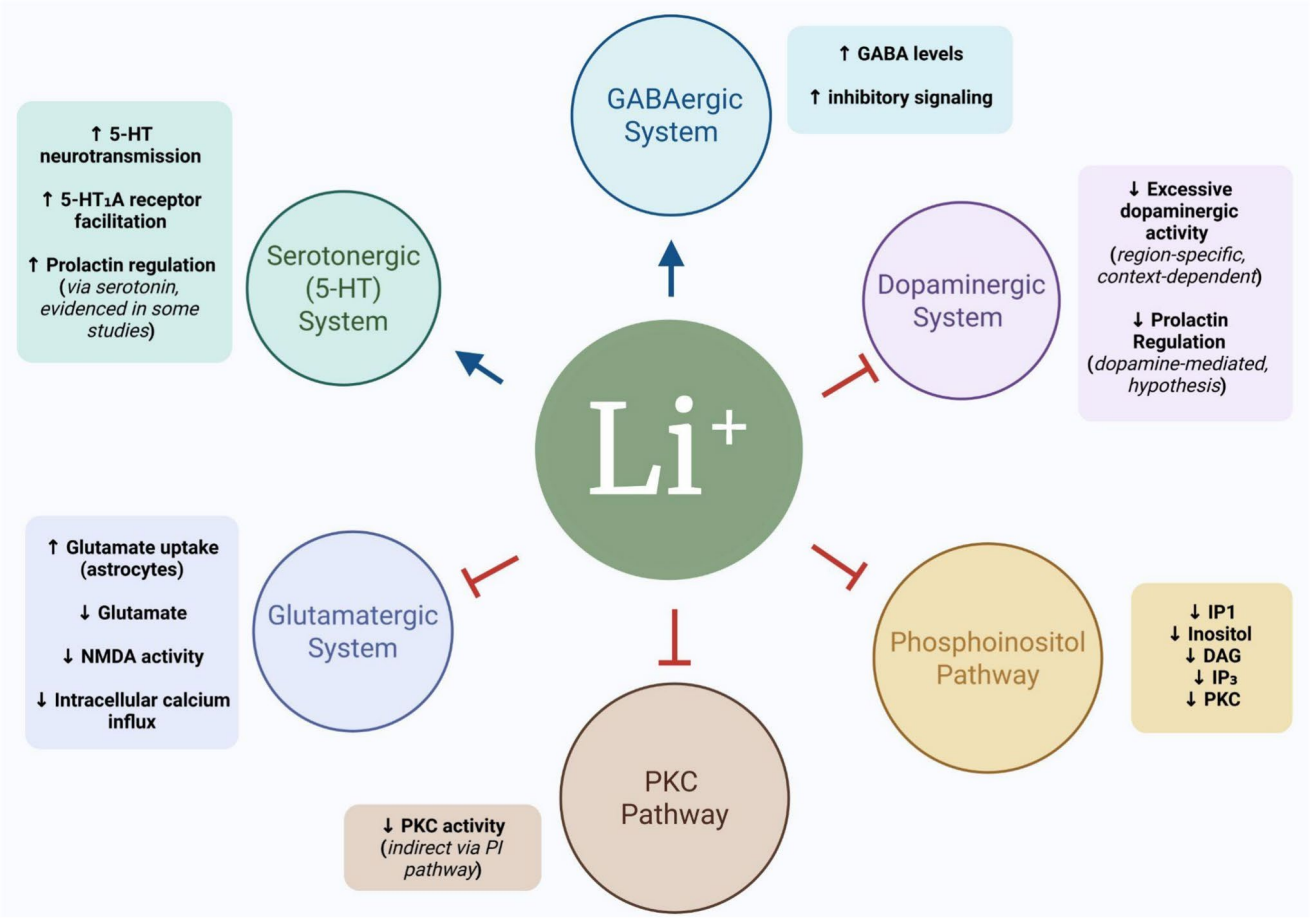
Lithium and Phosphoinositol pathway. Lithium intervenes in the signaling of inositol trisphosphate (IP3) and diacylglycerol (DAG) by inhibiting the conversion of inositol diphosphate (IP2) into inositol monophosphate (IP1) and subsequently into inositol. This process reduces free inositol and DAG, which serve as second messengers in numerous cascades of reactions, including the mobilization of intracellular calcium ions (Ca^2^ +) and the activation of Protein Kinase C (PKC). Created in BioRender

Lithium generally influences neurotransmitter systems, balancing excitatory and inhibitory neurotransmitters, increasing GABA/glutamate ratios, and acetylcholine/catecholamine activity to stabilize mood [58] (Fig. 2). Lithium generally reduces excitatory neurotransmission from dopamine and glutamate and increases inhibitory neurotransmission from GABA [72].

One of the mechanisms of lithium is to inhibit inositol phosphatase and thus reduce intracellular levels of inositol, a simple isomer of glucose that acts as a precursor to the second messenger system coupled to cholinergic, serotonergic, and adrenergic neurotransmitter systems. This is one of the hypotheses of the drug’s mechanism of action in the control of BD [26]. Inositol and lithium, as well as their combination, can attenuate adrenaline and dopamine release in the nucleus accumbens of rats with BD and in the in vitro evaluation of the tissue [6].

Lithium plays a pivotal role in regulating inositol trisphosphate (IP3) and diacylglycerol (DAG) signaling, acting as a modulator of this pathway. Several neurotransmitter systems utilize the phosphatidylinositol pathway via activation of G proteins. In this pathway, G protein activation stimulates the effector protein phospholipase C (PLC), which hydrolyzes a membrane phospholipid called phosphatidylinositol (PIP2), generating two critical second messengers: DAG and IP3. IP3 binds to a specific receptor located on the smooth endoplasmic reticulum, triggering the release of stored Ca^2^ + upon activation. DAG activates protein kinase C [62, 67].

Specifically, lithium acts by inhibiting the conversion of inositol diphosphate (IP2) to inositol monophosphate (IP1) and subsequently free inositol. This action significantly reduces free inositol and DAG concentrations, which serve as essential second messengers in various intracellular cascade reactions [14, 33]. These second messengers involve intricate biological processes, including the mobilization of Ca^2^ + within cells and the activation of PKC, a key enzyme in regulating multiple cellular signaling pathways. In individuals with BD, an increase in the concentrations of these substances is observed, contributing to imbalances in intracellular signaling pathways associated with disease symptoms [14, 33].

**Fig. 3:**
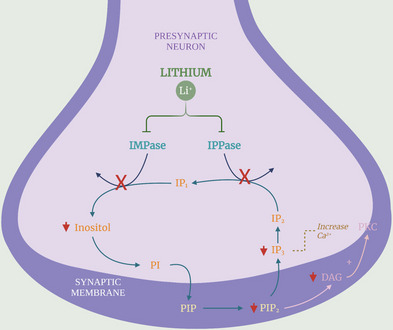
Effects of lithium on neurotransmitter systems and intracellular signaling pathways. Lithium exerts a modulatory effect on different neurotransmitter systems by enhancing inhibitory (GABAergic) and serotonergic neurotransmission while reducing excessive dopaminergic and glutamatergic activity. In the GABAergic system, it increases GABA levels and enhances inhibitory signaling. In the serotonergic system, lithium increases 5-HT neurotransmission, facilitates 5-HT1A receptor activity, and influences prolactin regulation. It also reduces dopaminergic hyperactivity, which may be context- and region-dependent, further contributing to prolactin regulation. In the glutamatergic system, lithium increases glutamate uptake by astrocytes, reduces synaptic glutamate levels, decreases NMDA receptor activity, and lowers intracellular calcium influx. Additionally, lithium inhibits the phosphatidylinositol (PI) signaling pathway, resulting in reduced levels of inositol, IP1, DAG, and IP3, which in turn leads to decreased protein kinase C (PKC) activity. This impacts intracellular cascades related to neuronal excitability and mood stabilization. Created in BioRender

The action of lithium in diminishing levels of free inositol and DAG holds significant therapeutic implications (Fig. 3). Lithium attenuates PKC activity and excessive calcium ion release by reducing these second messengers. This, in turn, helps modulate the cellular response to excitatory stimuli, contributing to emotional stability and a reduction in symptoms observed in patients with BD [14, 33].

GABA is an inhibitory neurotransmitter at suboptimal levels in cases of BD. It helps modulate the dopaminergic and glutamatergic systems; therefore, low levels of GABA are associated with high levels of excitatory neurotransmission. Lithium ensures adequate levels of GABA; with that, there is a reduction in the level of glutamate, which decreases the receptivity of N-methyl D-Aspartate (NMDA). The drug also blocks calcium influx after NMDA receptor activation [72].

The effect of lithium on the glutamatergic system has yet to be fully elucidated. However, it is possible to identify a wide range of changes in metabolites and neurotransmitters related to the system in patients with BD [60]. Studies indicate that chronic lithium administration facilitates the postsynaptic serotonin 5-HT1A receptor, which inactivates the NMDA glutamate receptor [72].

The glutamatergic system is essential for cognitive function and neuronal plasticity. Lithium modulates this system by increasing the capacity of the glutamate uptake transporter in the cerebral cortex and stabilizing synaptic concentrations of glutamate. Research suggests that this stabilization is a key component of lithium’s neuroprotective effect against excitotoxicity [100].

Acute use of lithium increases synaptic concentrations of glutamate by inhibiting neurotransmitter uptake, and chronic use leads to an increase and stabilization in the capacity of the glutamate uptake transporter in the cerebral cortex of mice with BD [34]. Chronic treatment may reduce excitatory neurotransmission and contribute to neuroprotective effects by increasing glutamate uptake and reducing the increase in intracellular calcium through the activation of NMDA receptors [58].

A study conducted with 19 individuals with BD type II and 17 controls found a varied effect of lithium on glutamate levels, depending on the administered dose of the drug. The analysis was performed by estimating the partial correlation of the volume identified in 3 T proton magnetic resonance spectroscopy. A sub-dose of lithium at 0.2 to 0.49 mmol/L decreased glutamate concentrations, and the standard dose (≥ 0.50 mmol/L) increased glutamate concentrations over time [120].

The research was conducted in healthy adults who were administered lithium subchronically and evaluated its effect using proton magnetic resonance spectroscopy. After two weeks of lithium use, glutamine significantly decreased in the left basal ganglia. It showed a decreasing trend in the right basal ganglia, glutamine–glutamate decreased in the right basal ganglia, and a decreasing trend in the left basal ganglia, and glutamate and GABA showed no changes. The researchers hypothesized that the decrease in glutamine–glutamate levels is associated with the pharmacologic actions of subchronic lithium treatment [100].

Other systems modulated by lithium are dopaminergic and serotonergic. These systems have distinct roles in prolactin release, with the dopaminergic system inhibiting its release and the serotonergic system stimulating it. Prolactin is a stress hormone. Therefore, its release is influenced by different stimuli, in addition to being influenced by the circadian cycle. Studies on the effect of lithium on prolactin levels are controversial. Studies have hypothesized that treatment with lithium can increase dopamine synthesis and the activity of dopaminergic neurons, culminating in inhibitory control of prolactin secretion. Still, there need to be studies that elucidate this hypothesis. On the other hand, research reports that lithium increases serotonergic neurotransmission in the CNS and thus increases prolactin release. In addition, the serotonergic system inhibits the dopaminergic system in some brain regions, which also influences prolactin release [10].

A survey analyzed the serum level of prolactin in individuals treated with lithium compared to individuals treated with lithium and additional medication. It did not identify any difference in the levels of the substance between the groups. However, the authors suggest that further studies be conducted regarding the effect of lithium on prolactin levels, considering the findings on the drug’s function in the serotonergic and dopaminergic systems and its relationship with the hormone [78].

Several studies indicate the effect of lithium on serotonergic neurotransmission. The drug stimulates serotonin neurotransmission and thus has a positive impact even in individuals with depression resistant to treatment with tricyclic antidepressants. This effect may have occurred because tricyclic antidepressants sensitize neurons to serotonin, and lithium increases the activity of serotonin-containing neurons [29]. The specific mechanism by which this happens could be more precise, but other studies report the influence of the serotonergic system on lithium.

A study in Germany found that altering the gene that encodes the serotonin transporter, known as 5-HTT, can influence an individual's response to lithium. Specifically, the 5-HTTLPR polymorphism was addressed as an alteration that increases the drug’s effect on individuals and, therefore, a beneficial change for patients using lithium [103]. Other studies related to pharmacogenetics are addressed in topic 4 of this article, called Pharmacogenetics of Lithium.

## Lithium and the Hypothalamic–Pituitary–Adrenal Axis

**Fig. 4:**
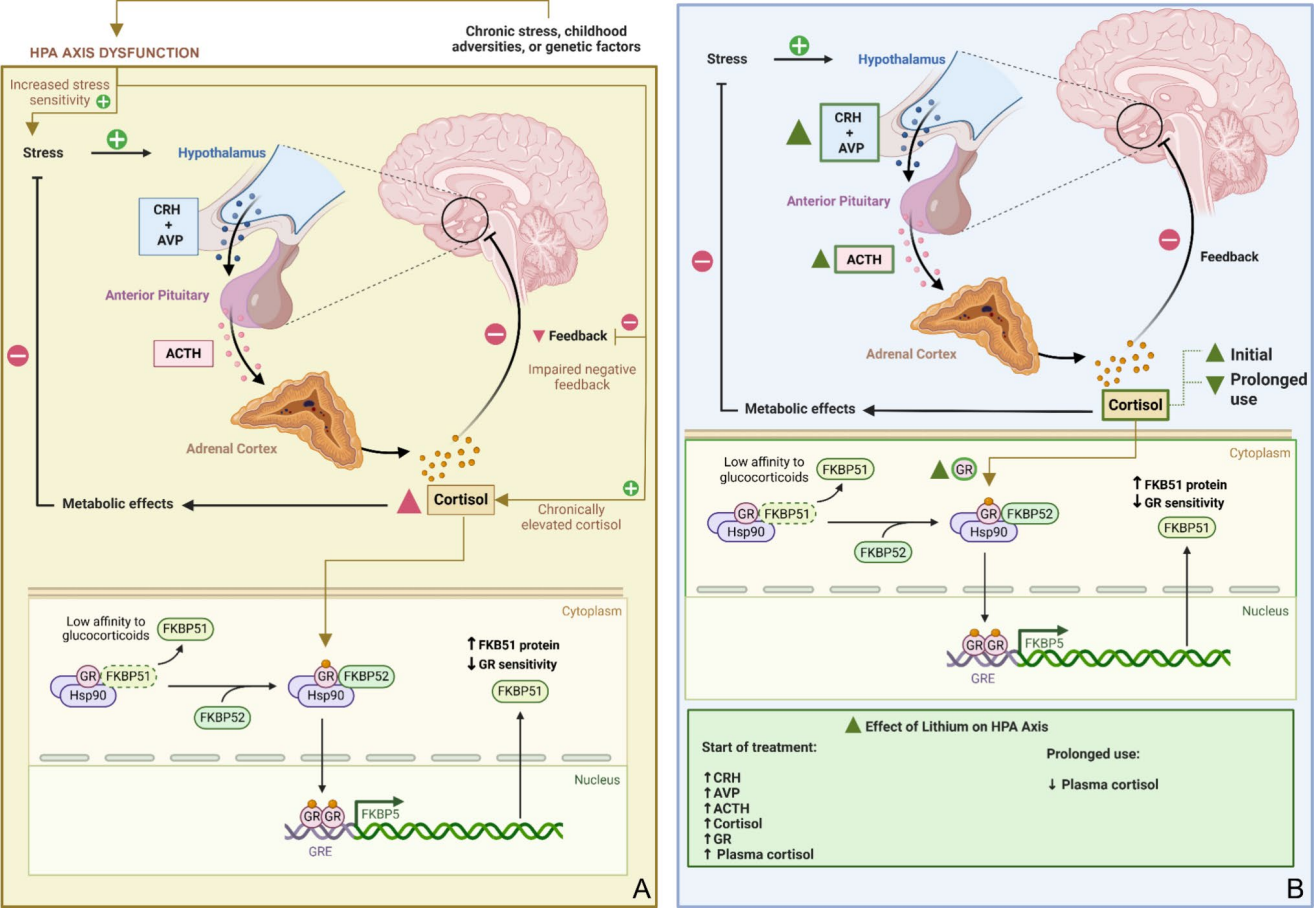
**A** Representation of HPA (hypothalamic–pituitary–adrenal) axis dysfunction, commonly associated with chronic stress, childhood adversity, or genetic factors. Increased stress sensitivity leads to hypothalamic secretion of CRH (corticotropin-releasing hormone) and AVP (arginine vasopressin), which stimulate the anterior pituitary to release ACTH (adrenocorticotropic hormone). ACTH acts on the adrenal cortex, inducing cortisol release. Impaired negative feedback results in chronically elevated cortisol levels. In the cytoplasm, the glucocorticoid receptor (GR) forms a complex with the chaperone Hsp90 (heat shock protein 90) and the co-chaperone FKBP51, which reduces GR affinity for glucocorticoids. Activation requires the replacement of FKBP51 by FKBP52, allowing GR nuclear translocation, dimerization (GR–GR), and binding to glucocorticoid response elements (GREs) in the DNA, thereby regulating gene expression. HPA axis dysfunction increases FKBP51 expression and decreases GR sensitivity, perpetuating impaired feedback. **B** Effects of lithium on the HPA axis and GR signaling. At treatment initiation, lithium increases CRH, AVP, ACTH, cortisol, GR expression, and plasma cortisol, indicating HPA axis activation. Prolonged treatment reduces plasma cortisol, suggesting a modulatory effect. Lithium also modulates the FKBP51/FKBP52 balance, favoring GR activation and restoring receptor sensitivity. These effects contribute to the partial reestablishment of cortisol negative feedback and stress response modulation. Created in BioRender

The Hypothalamic–Pituitary–Adrenal (HPA) axis has a stress response function and acts by triggering a cascade of reactions in the CNS from the release of glucocorticoids [92] (Fig. 4).The effect of the axis is essential to guarantee survival, but the cessation of the cascade of reactions is necessary when the triggering situation ends. Physiologically, the HPA axis stops releasing glucocorticoids due to negative feedback; however, dysfunctions in this system culminate in the release of high levels of glucocorticoids, which negatively influence mental health and increase the risk of developing disorders [61].

Dysregulation of the HPA axis contributes to the pathophysiology of mood disorders, including BD [117]. Although the exact mechanism by which this occurs is not well established, research indicates that genetic issues, childhood adversities, and stressful events in adult life interfere with the function of the HPA axis. As a result, there is an increased risk of individuals developing mood disorders [116].

The combined dexamethasone/corticotropin-releasing hormone (DEX/CRH) test is used in several studies to identify HPA dysfunction [18]. Individuals with BD have an increased cortisol response to the DEX/CRH test compared to controls. This alteration is identified in patients in remission and those not [116].

Lithium interferes with the HPA axis by enhancing the release of corticotropin-releasing hormone (CRH), vasopressin (AVP), adrenocorticotropic hormone (ACTH), cortisol, and glucocorticoid receptor (GR) messenger RNA. At the beginning of treatment, the drug induces an increase in plasma cortisol, but with prolonged treatment, these plasma cortisol levels tend to decrease [18, 117].

One study found that increasing the dose of Lithium increases the ACTH and cortisol response, as verified by the combined DEX/CRH test [61]. Another study, using the dexamethasone suppression test, demonstrated that increasing the Lithium dose induces an increase in cortisol post-dexamethasone [19]. Both results suggest that one of the mechanisms underlying the drug’s function involves modulation of the HPA axis [17, 18].

Although several studies indicate that lithium interferes with the function of the HPA axis, the mechanisms by which this influence occurs are still unclear. Therefore, further research is needed to determine the pathways interconnected with the drug’s function in the HPA axis, which is highly related to the pathophysiology of several mental disorders, such as BD, anxiety, and depression.

## Lithium and Oxidative/Nitrosative Stress

Numerous studies have conclusively demonstrated that the production of reactive oxygen species (ROS) is pivotal in the pathophysiology of various neuropsychiatric disorders [13, 21, 74]. In physiological conditions, ROS are continuously generated and effectively controlled by intracellular and extracellular antioxidant systems [55]. Oxidative stress arises when oxygen-free radical production surpasses the body's antioxidant capacity. Oxygen-free radicals are oxygen-based chemical intermediates with high reactivity, and the balance between ROS production and processes aimed at reducing ROS is referred to as the redox state [98].

ROS can be generated in different compartments of a mammalian cell, with mitochondria being the primary source responsible for producing the majority of ROS. Other sources, such as monoamine oxidase (MAO) and nitric oxide synthase (NOS), contribute significant quantities of ROS, playing roles in various cellular physiopathological processes. Studies have demonstrated that ROS plays a critical role as an essential signaling molecule in the proper induction of synaptic plasticity and memory formation [74]. However, in situations where the generation of free radicals exceeds the antioxidant defense capacity, oxidative stress can lead to membrane degradation, cellular dysfunction, and apoptosis [105].

Therefore, ROS can be considered physiological mediators of great importance in plasticity and signaling, but they can become detrimental to neuronal function when they accumulate excessively in the brain [74]. The brain is particularly susceptible to oxidative damage among the body's organs due to its significant oxygen demand [51, 52]. Oxidative damage can occur due to increased ROS production and decreased repair or removal processes, such as failing to eliminate oxidized proteins or repair oxidized DNA quickly enough [79].

## Lithium and Mitochondrial Respiratory Chain Function

Mitochondrial dysfunction in the CNS is a pathogenic pathway for various disorders associated with progressive atrophic/degenerative changes. Long-term lithium treatment can improve the rate of cellular respiration and mitochondrial function as determined by mitochondrial membrane potential and mitochondrial oxidation in SH-SY5Y cells. Furthermore, in this study, the long-term lithium treatment protected against methamphetamine (Meth)–induced toxicity at the mitochondrial level. These agents also prevented the Meth–induced reduction of mitochondrial cytochrome c, the mitochondrial anti-apoptotic Bcl-2/Bax ratio, and mitochondrial cytochrome oxidase (COX) activity [7].

Lithium has protective functions in human cells by blocking the tyrosine phosphorylation of the NMDA receptor subtype 2B and preventing the massive influx of Ca2 +. Lithium levels in patients with BD during depressive episodes are positively correlated. Thus, it is inferred that lithium increases mitochondrial respiration, decreases the pro-apoptotic enzyme glycogen synthase kinase-3, and inhibits the expression of the pro-apoptotic genes Bax and p53. The same protective effect against Ca2 + overload was also observed in the brain mitochondria of rats, where lithium increased the activity of mitochondrial complexes I, II, and III [68]. On the other hand, another study involving mitochondrial respiratory chain stabilizers for D-amphetamine-induced BD reported that the results demonstrated the use of lithium reversed and prevented d-AMPH-induced hyperlocomotion, but did not alter the mitochondrial respiratory chain complexes I, II, III, and IV [109].

Excessive glutamate signaling can induce neuronal dysfunction and cell death through the production of excitotoxicity, a process characterized by a large influx of calcium, which results in a cascade of events involving the disruption of calcium-dependent cellular pathways, mitochondrial dysfunction, the production of oxidative stress, and the activation of apoptosis. Glutamate signaling occurs through ionotropic or metabotropic glutamate receptors. The NMDA receptor is an ionotropic glutamate receptor that causes a calcium influx upon activation. It gives it convulsant properties at high doses due to a dramatic increase in action potentials, which can be observed as spikes in EEG recordings. In this study involving chronic excessive activation of NMDA receptors to determine whether it would trigger mitochondrial ETC malfunction and lipid peroxidation in the brain, a reduction in complexes I and III was identified, where lithium was unable to protect these transmembrane proteins in the frontal cortex, where higher oxidative stress and ATP reduction were observed [59].

In an assessment of leukocyte mitochondrial complex I activity in BD during depressive episodes after lithium treatment, a significant decline in depressive symptoms and manic episodes was observed throughout the treatment, with no statistical differences detected between male and female patients. Furthermore, lithium treatment significantly increased complex I activity; however, the activities of complexes II, III, and IV did not show any alterations. Despite the differences in complex activities, the mitochondrial electron transport chain (ETC) activity did not show any difference in BD patients during depressive episodes compared to healthy controls. Complex I activity may be selectively altered in later stages of BD when increased oxidative stress is more prominent, as elevated oxidative stress leads to ETC dysfunction, generating more oxidative stress and creating a vicious cycle [31].

In a review study on the role of heavy metals in induced neurotoxicity, Vellingiri et al. [112] describe how lithium may be related to aging processes that involve protein secretion, ultimately resulting in neurodegenerative diseases such as Parkinson’s Disease (PD). Based on preclinical models and theoretical reviews, lithium is often described as a neuroprotective agent that regulates autophagy. However, some laboratory studies suggest lithium might increase protein aggregation in certain neurodegenerative contexts or cause mitochondrial degradation through autophagy. These findings currently lack clinical confirmation in human trials. In PD, autophagy is enhanced via the mTOR pathway, resulting in the restriction of GSK-3β. Lithium directly or indirectly inhibits GSK-3β, which functions in cell death, cell cycle, and carcinogenesis, and is a vital regulator of many signaling pathways. The accumulation of α-synuclein (αSyn) leads to impaired autophagy and lysosomal function, which is structurally related to phosphomonoesterases and GSK-3β. Thus, lithium toxicity can occur when high doses are administered or treated over two decades [112].

Cycloheximide and lithium chloride are identified as effective inhibitors of autophagy. The study reveals that rotenone induces cell death through the activation of autophagy, while cycloheximide prevents this activation. Other experiments with cell death inhibitors indicate that only the autophagy inhibitor rescues cell survival. Lithium chloride also effectively preserves cell viability, suggesting a possible relationship between autophagy and cell function. The combination of autophagy and translation inhibitors, such as 3-methyladenine and lithium chloride, shows synergistic effects in protecting cell viability and mitochondrial respiratory capacity, suggesting beneficial implications in RC disease [87].

Variables such as patient age, disease course, onset of the disease, and genetic factors influence the accumulation of lithium in erythrocytes of manic-depressive patients. It was observed that patients who respond well to lithium treatment are more likely to come from families with a higher prevalence of BD and respond more favorably to lithium. These findings align with previous research on BD, which highlighted mitochondrial dysfunction and its stronger correlation with neurons in genetically lithium-responsive patients [102].

In a study on mitochondrial dysfunction and its role in various health conditions, including neurological disorders, diabetes, and cancer, the effects of different compounds such as lithium salts, trehalose, rapamycin, resveratrol, N-acetylcysteine, and Mn-Tbap on mitigating mitochondrial damage caused by rotenone exposure were investigated. The results indicated that lithium positively affected normalizing mitochondrial respiration and cell viability, especially when added after 48 h of rotenone exposure for 96 h. Substances such as trehalose, rapamycin, and resveratrol also exhibited effects, but these varied across different parameters and rotenone exposure conditions. The study suggests that these compounds may be considered adjuncts to lithium treatment, highlighting the need for further investigation into the underlying mechanisms involved [28].

Regarding oxidative stress associated with mitochondrial complex I dysfunction and its role in BD, the importance of glutathione, an endogenous antioxidant, in preventing oxidative damage has been highlighted. The study investigated whether lithium, a mood stabilizer, can prevent oxidation and nitration of proteins caused by mitochondrial dysfunction by increasing glutathione levels. The results indicated that lithium prevents the decrease in complex I activity and cell viability and reduces protein carbonylation and nitration. However, the relationship between lithium and glutathione varies across different protein modifications, suggesting the complexity of lithium’s antioxidant mechanisms. The study highlights the significance of these findings in developing targeted therapeutic interventions to combat oxidative stress associated with BD [80].

Lithium is crucial in various biological processes, particularly in the treatment of BD. In a study that reviewed transcriptomic, proteomic, and metabolomic research to understand the effects of lithium at different biological levels, regulated patterns by lithium were identified in genes, proteins, and metabolites, addressing areas such as neuroprotection, oxidative stress, energy production, mitochondrial function, and impact on Alzheimer’s disease (AD). The review emphasized the significance of lithium in various biological processes. Additionally, it highlights the importance of considering environmental issues associated with industrial lithium waste, given its widespread use in modern society [93].

## Lithium, Inflammation, Neuroinflammation, and Immune Functions

**Fig. 5:**
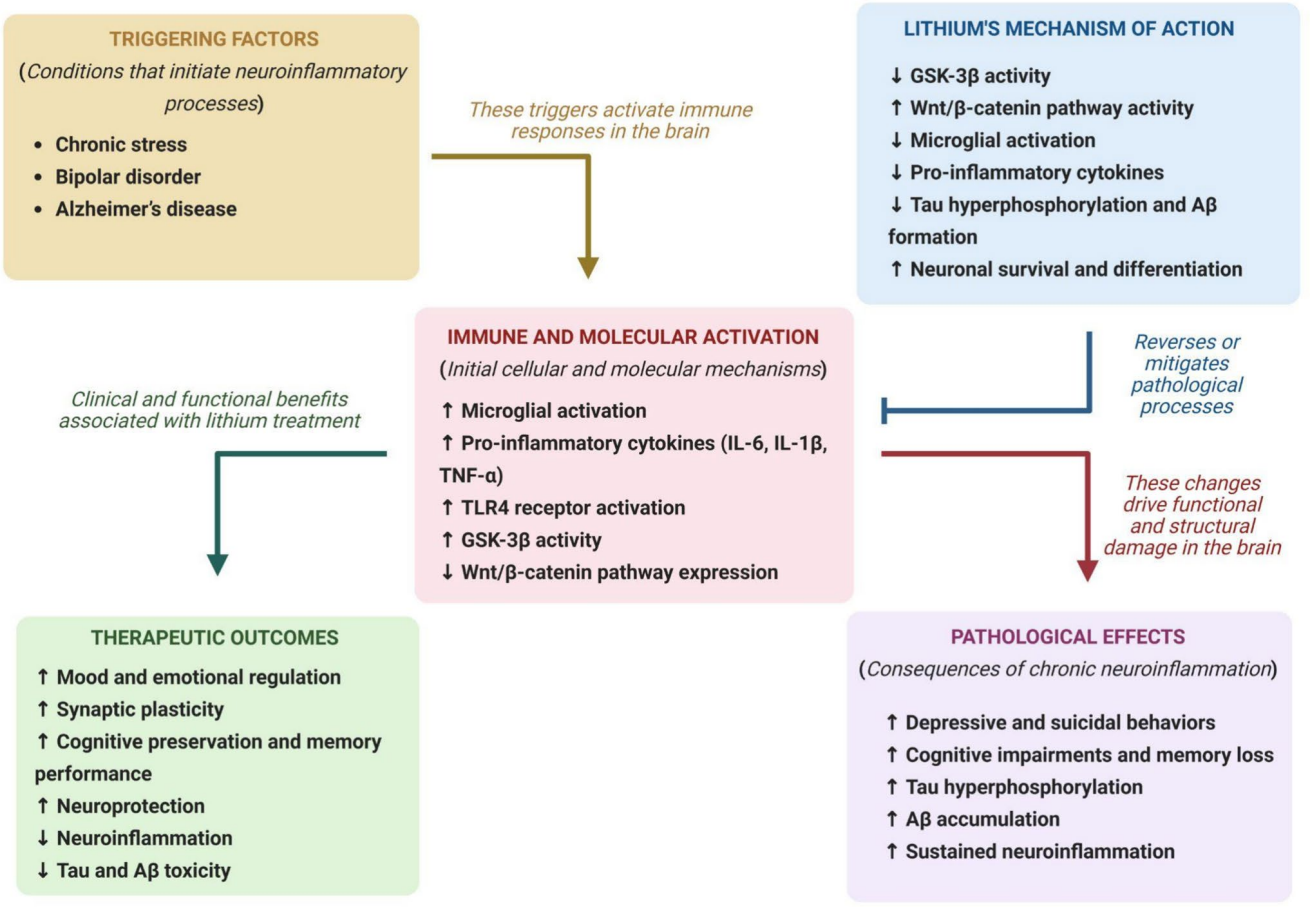
Lithium’s Modulatory Effects on Neuroinflammatory Pathways and Clinical Outcomes. The figure depicts a cascade of events triggered by factors such as chronic stress, bipolar disorder (BD), and Alzheimer’s disease (AD), which activate brain immune pathways leading to microglial activation and release of pro-inflammatory cytokines (IL-6, IL-1β, TNF-α), activation of TLR4 receptors and GSK-3β, and suppression of the Wnt/β-catenin signaling pathway. This immune/molecular response results in pathological outcomes including cognitive dysfunction, suicidal behavior, sustained neuroinflammation, and deposition of hyperphosphorylated Tau and Aβ proteins. Lithium treatment modulates these pathways by inhibiting GSK-3β, suppressing microglial activation and inflammatory cytokine release, restoring Wnt/β-catenin signaling, and promoting neuronal survival. These effects lead to the reversal of pathological processes and therapeutic benefits, such as improved emotional regulation, cognitive preservation, neuroprotection, and a reduction in neuroinflammation and Tau/Aβ toxicity. Created in BioRender

Lithium has modulatory effects on neuroinflammatory pathways and influences clinical outcomes (Fig. 5). In a study investigating the expression of the Wnt/ß-catenin pathway in the hippocampus of rats exposed to chronic mild stress (CMS), the effects of lithium on this expression were evaluated. Chronic stress-induced depressive behavior, pro-inflammatory microglial activation, and reduced expression of the Wnt/ß-catenin pathway in the hippocampus. Chronic lithium treatment improved behavior, reduced microglial activation, and increased expression of the Wnt/ß-catenin pathway. The results suggest that lithium’s inhibition of GSK-3ß may prevent pro-inflammatory microglial activation associated with depression, highlighting the Wnt/ß-catenin pathway as a potential therapeutic target for various neurological disorders [49].

Stress is identified as a common precursor to suicidal behavior and is associated with inflammation. Different types of psychological stress can activate inflammatory responses involving inflammatory cytokines, and this inflammation may play a significant role in triggering suicidal behaviors. Toll-like receptor 4 (TLR4) is a crucial component in the stress response, activating the immune system using pathogen-like mechanisms to induce an inflammatory response, and TLR4 is expressed in various cells, including microglia, astrocytes, and neurons. Thus, there appears to be a link between elevated levels of inflammatory cytokines such as interleukin-6 (IL-6) and suicidal behaviors. Additionally, associations are mentioned between inflammation and aggressive, impulsive, and depressive behaviors. Lithium’s ability to reduce aggressive and suicidal behaviors is closely linked to its anti-inflammatory properties. As previously detailed, lithium-mediated inhibition of GSK-3 effectively suppresses the inflammatory responses triggered by stress [16].

In an animal model of dementia using Wistar rats, the therapeutic potential of lithium and its association with memantine was evaluated. The study found that in these rats, the combination reduced spatial memory loss and neuroinflammation induced by Aβ1–42 oligomers. While promising, these results are limited to preclinical observations. Both medications individually improved spatial memory deficits, but the combination provided superior results. Regarding neuroinflammation, both lithium and memantine reduced IL-4 levels in the frontal cortex. In the hippocampus, only memantine and the combination of lithium and memantine were effective. The treatments also led to reductions in IL-1b levels in the frontal cortex and hippocampus, as well as decreased levels of TNF-α in the hippocampus. Thus, both lithium and memantine may represent potential therapy against cognitive impairment and Ab1-42-induced neuroinflammation, suggesting their combination could be a promising alternative in treating Alzheimer's disease-like dementias [20].

In this study, cognitive dysfunction and depressive psychopathology were related, highlighting cognitive deficits associated with depression through assessment of pathophysiological mechanisms, particularly those related to neuroinflammation, emphasizing the influence of hyperphosphorylated tau in AD pathology and stress-related depressive disorders. Lithium was used to inhibit GSK-3ß, and the impact of this process on neuroinflammation and tau deposition was examined. Lithium improved depressive and cognitive deficits, reduced GSK-3ß expression and tau hyperphosphorylation, prevented neuroinflammation, and increased neuronal survival. The study highlights the adaptive capacity of prior immune challenges to mitigate inflammatory damage and preserve cognitive abilities, while also underscoring the beneficial effect of lithium in preventing tauopathy and neuroinflammation. However, it emphasizes the need for further research to understand better the complex interactions between immune activation and chronic stress exposure. The study provides insights into the neurobiological mechanisms underlying depression and cognitive dysfunction, suggesting possible therapeutic strategies [35].

Cognition plays a central role, with severe cognitive deficits present in 25% of patients, contributing to disease-associated disability. BD is a chronic disorder affecting approximately 1–5% of the population. The alternation between opposite mood states, mania and depression, is the hallmark of BD. Neuroinflammation is associated with the pathophysiology of BD, characterized by elevated levels of pro-inflammatory cytokines in patients. Studies suggest a link between neuroinflammation and stimulant-induced manic-like behavioral changes, such as with amphetamine. Building on this principle, studies have explored the therapeutic potential of doxycycline (DOXY)—a second-generation tetracycline—in BD treatment. Preclinical experiments in mice indicate that DOXY, alone or combined with lithium, reverses behavioral and neuroinflammatory changes induced by amphetamine. The clinical relevance of this adjunctive therapy in humans remains to be established. DOXY also exhibits antioxidant effects, protecting against oxidative damage in the brain. Results suggest that DOXY may be a promising adjunctive therapy for BD, reversing cognitive deficits and amphetamine-induced neuroinflammation in mice. Microglial activation and inhibition of GSK3ÿ kinase appear to be involved in DOXY’s therapeutic effects [25].

Regarding postoperative cognitive dysfunction (POCD)—a clinically significant syndrome associated with postoperative complications and increased morbidity and mortality—neuroinflammation is suggested to play a role in its pathogenesis. Based on this principle, a study evaluated the effects of prophylactic lithium treatment on cognitive dysfunction, as it is recognized as a neuroprotective agent that could positively influence this response. It was found that splenectomy in elderly rats resulted in systemic, central, and hippocampal inflammation, along with cognitive deficits. Prophylactic lithium treatment showed beneficial effects by reversing hippocampal microgliosis, inhibiting pro-inflammatory cytokines, and improving mental performance. Inactivation of TLR4 signaling is a crucial mechanism underlying lithium’s protective effects, as it inhibits both systemic and hippocampal inflammation [66].

## Lithium, Intracellular Signaling and Brain Plasticity

According to this study, lithium has been shown to restore neurotransmission and brain structure, suggesting the existence of standard dysfunctional molecular and morphological mechanisms between psychiatric and neurological disorders. The study highlights lithium’s efficacy in treating typical BD, especially in episodes of mania or hypomania, focusing on neural adaptations associated with stimulant-induced psychotic development and exploring, through sensitization processes, shared characteristics between BD and other psychiatric disorders such as schizophrenia. The multiple functions of lithium underscore its exceptional pharmacology, suggesting that these actions can guide multi-drug strategies. Despite limitations, such as the lack of in-depth analyses on how these mechanisms contribute to neurodegeneration in different neuronal populations and brain areas, the article highlights lithium’s exceptional pharmacology. It suggests that its actions can guide more effective therapeutic strategies and multi-drug developments [90].

In a review study examining the role of lithium in the pharmacotherapy of BD with a focus on therapeutic, neurobiological effects, and underlying mechanisms, lithium salt was highlighted as fundamental in maintenance therapy and acute manic episodes in BD, as well as playing a significant role in preventing suicide and unipolar depression. The work explored the biological models underlying stress, resilience, and homeostasis, identifying lithium’s regulatory targets at different biological levels and discussing its influence on brain structure, neurochemistry, epigenetics, stress pathways, and inflammation. Biomarkers, such as brain imaging studies, are reviewed, showing that lithium can reverse structural brain changes associated with BD. The effects of lithium on glutamate levels, mitochondrial function, and neurotrophic factors are discussed as part of its mechanisms of action. The role of lithium in regulating biomarkers such as GSK-3β and AKT, as well as other intracellular signaling modulators, was identified and associated with blood lithium levels, particularly in relation to efficacy and adverse effects. This raises the question of whether lower lithium levels might better prevent adverse effects and improve adherence without compromising clinical outcomes [69].

Currently, there is a strong association of BD with neuroplasticity disorders. Based on this principle, a study aimed to understand the neurobiological substrate of BD, as well as the mechanisms of action of medications such as lithium and aripiprazole in animals. The study used rats’ intracranial self-stimulation (ICSS) paradigm to assess the acute and chronic effects of lithium and aripiprazole on brain reward systems. Additionally, it examined molecular markers of neuroplasticity in specific regions of the limbic forebrain. The results revealed that chronic administration of lithium induced tolerance to its anhedonic effect, while aripiprazole showed sustained anhedonic effects. The study suggests that although lithium and aripiprazole share some common intracellular effects, lithium has a broader and more robust impact, which may contribute to the observed differences in behavioral responses between the two drugs in the chronic treatment of BD [75].

Clinical evidence indicates that lithium may have neuroprotective effects, especially in patients with BD undergoing long-term treatment and in AD. This study hypothesized that lithium benefits by stabilizing intracellular calcium, antagonizing NMDA receptors, and inhibiting IMP. The calcium hypothesis in AD proposes that elevated intracellular Ca2 + concentrations are associated with the disease. Lithium, at therapeutically relevant concentrations, has been experimentally shown to reduce intracellular Ca2 + levels. Additionally, it acts by inhibiting NMDA receptors and IMP, which may contribute to stabilizing intracellular Ca2 +. The authors suggest that although some evidence suggests that the enzyme GSK-3 may be involved in AD, lithium’s effect on intracellular Ca2 + appears to be more pronounced than its influence on GSK-3. Thus, lithium may prevent AD by stabilizing intracellular Ca2 + through NMDA receptor antagonism and IMP inhibition. Researchers also highlight the hypothesis of calcium dysregulation in AD, suggesting that controlling intracellular Ca2 + levels may delay or prevent disease progression. Moreover, there is a suggestion that an age-related decrease in cellular energy may predispose neurons to disruptions in Ca2 + homeostasis, and subtherapeutic doses of lithium could attenuate these disturbances, offering a potential therapeutic strategy to modify disease progression in AD [114].

The study examines the impact of mood-stabilizing medications, including lithium, valproate (VPA), carbamazepine (CBZ), and lamotrigine (LTG), on dendritic growth and synaptic protein expression in primary hippocampal neurons. The results indicate that all medications significantly promote dendritic growth, but their mechanisms of action differ. Lithium and valproate exert neurotrophic effects that are blockable by specific inhibitors, whereas carbamazepine and lamotrigine are not affected by the same inhibitors. Additionally, lithium, valproate, and carbamazepine showed neuroprotective effects, preventing reductions in synaptic protein levels induced by cytotoxicity. However, lamotrigine did not exert this neuroprotective effect. These results suggest that these medications may positively influence neural plasticity and protect against cellular damage in the hippocampus, but their mechanisms and efficacy vary. The study also examined the effects of these mood-stabilizing medications on hippocampal dendritic growth and analyzed whether the PI3K, ERK, and PKA signaling pathways are responsible for their neurotrophic effects. The findings indicated that lithium, valproate, and carbamazepine promoted dendritic growth in hippocampal neurons, and these effects are mediated through the PI3K, ERK, and PKA signaling pathways. Moreover, these drugs exhibit neuroprotective effects by increasing synaptic protein expression under toxic conditions induced by B27 deprivation. These discoveries contribute to a deeper understanding of the underlying mechanisms of these mood-stabilizing medications in the context of BD [30].

In this study on cellular aging and its associations with cellular degeneration, particularly in age-related diseases such as AD, the effects of chronic treatment with therapeutic concentrations of lithium on SH-SY5Y cells were investigated over more than ten months. The analysis revealed that lithium influenced cell morphology, increasing cell density and promoting the growth and branching of neurites, which resulted in the formation of neuronal networks. Additionally, the study examined changes in the cytoskeleton, highlighting the organization of microtubules and neurofilaments. It was demonstrated that lithium affected cytoskeletal organization, enhancing neurite outgrowth, growth cones, and synapses without causing significant alterations in microtubules. Treatment with lithium also influenced the expression of genes related to cytoskeletal modulation. When examining GSK3, researchers observed that lithium did not induce significant changes in GSK3 expression and activity, suggesting that the mechanism of action of lithium in this context may be independent of direct GSK3 inhibition. Thus, prolonged treatment with therapeutic lithium doses was associated with changes in cytoskeletal proteins, promoting neuritogenesis in SH-SY5Y cells [81].

In this study, the authors emphasize the importance of understanding the molecular pathways underlying the effects of mood-stabilizing medications such as lithium and valproic acid in the treatment of BD and major depression. Post-mortem and brain imaging studies have revealed structural alterations in these conditions. Mood stabilizers, along with antidepressants and electroconvulsive therapy, have been associated with activating intracellular signaling pathways that promote neurogenesis and synaptic plasticity. BD is discussed in terms of its heterogeneity and heritability, with genetic studies identifying loci associated with bipolar risk. Structural brain changes, such as reductions in prefrontal cortex and hippocampal volume, are observed in BD and major depression. In this context, mood-stabilizing medications are analyzed for their impact on intracellular signaling pathways that promote neurogenesis and synaptic plasticity, particularly through the regulation of neurotrophic factors like BDNF [27].

The research findings on the effects of Lithium are detailed in Table 1 (human studies), Table 2 (animal models), and Table 3 (in vitro models).

**Table 1:** Studies of the Effects of Lithium on the BD I and BD II Patients and in Other Psychiatric Disorders

Patients	Behavioral Therapeutic Response	Main physiological findings	Reference
284 patients with BD and 295 controls	CC genotype of the rs2769605 variant of the NTRK2 gene is associated with decreased response to lithium in people with BD I; AA genotype of the rs1565445 variant is not associated with response to lithium	Polymorphisms in the NTRK2 gene influence lithium effectiveness	[115]
138 patients with BD I and 131 controls	The AA genotype of the rs334558 variant of the GSK3B gene is associated with decreased response to lithium in individuals with BD I treated for at least 24 months	Lithium inhibits the brain activity of GSK-3 and signal transduction	[63]
282 patients with BD I or II	GG genotype of the rs6438552 variant of the GSK3B gene is associated with increased response to lithium in people with BD; the T allele of the rs2071427 variant of the NR1D1/THRA gene is also associated with increased response to lithium	Lithium inhibits the brain activity of GSK-3	[76]
2563 patients with BD	Increased response with certain genotypes	rs78015114, rs79663003 T allele, and rs74795342 G allele associated with increased response	[53]
2043 patients with BD type I and 543 with BD II	Decreased response with specific genotypes	Polymorphisms in ADCY1, FAM177A1, MYO1H, FAM178B, GRAMD1B linked to decreased response; ZNF804A variants linked to increased response	[3]
52 patients with BD	Increased response with certain genotypes	OR52E2, OR52J3, and ASIC2 gene variants influence response	[101]
360 patients with BD I or II	More effective in BD II	Higher efficacy for BD II in a one-year longitudinal study	[107]
57 patients with BD	Improved mood regulation and neuroprotection	Lithium increases grey matter volume and enhances plasticity and resilience	[12]
42 patients with BD	Neuroprotection and reduced depressive symptoms	Lithium inhibits GSK3β expression	[56]
2586 patients with BD	Patients with BD and lower genetic susceptibility to major depression are more likely to respond well to lithium treatment	Higher polygenic load for major depression is associated with a less favorable response to lithium treatment in patients with BD	[2]
19 individuals with BD type II and 17 controls	Glutamate concentrations in the hippocampus demonstrated a bimodal response to lithium plasma levels, with sub-dose levels (0.2 to 0.49 mmol/L) associated with decreased concentrations and standard dose (≥ 0.50 mmol/L) linked to increased concentrations over time	Glutamate concentrations in the hippocampus showed a bimodal response to lithium plasma levels, indicating that lower levels were associated with decreased concentrations, while standard doses were linked to increased concentrations over time	[120]
35 patients with BD	Potential increase in dopamine synthesis and activity	Lithium treatment may increase dopamine synthesis and activity of dopaminergic neurons, potentially inhibiting prolactin secretion and increasing serotonergic neurotransmission	[10]
39 patients with BD	Improvement in depression resistant to tricyclic antidepressants	Lithium stimulates serotonin neurotransmission, enhancing the effect of tricyclic antidepressants by sensitizing neurons to serotonin	[29]
50 patients with depression	Potential improved response to lithium treatment	Alteration in the gene encoding the serotonin transporter (5-HTTLPR polymorphism) may increase the drug's effect on individuals	[103]
21 patients with BD I and 3 with BD II	Reduction of inositol levels	Inhibits inositol phosphatase, reducing intracellular inositol levels, affecting cholinergic, serotonergic, and adrenergic neurotransmitter systems	[26]
23 patients with depression, 41 patients with BD, and 18 controls	1. Increased risk of mood disorders 2. Modulation of HPA axis	1. Dysregulation of the HPA axis contributes to mood disorders, influenced by genetics, childhood adversities, and adult life stressors 2. Lithium enhances the release of CRH, AVP, ACTH, cortisol, and GR mRNA; it initially increases plasma cortisol but decreases with prolonged treatment	[117]
30 patients with BD	Increased ACTH and cortisol response	Increasing the lithium dose increases the ACTH and cortisol response, as verified by the DEX/CRH test; the dexamethasone suppression test shows increased cortisol post-dexamethasone with higher lithium dose	[17, 18]
25 patients with BD during depressive episodes	Significant decline in depressive symptoms and manic episodes over time	Increased activity of mitochondrial complex I; no changes in complexes II, III, and IV; potential selective alteration of complex I activity in later stages of BD	[32]

ADCY1: Adenylate Cyclase 1, ACTH: Adrenocorticotropic Hormone, ASIC: Acid Sensing Ion Channel, AVP: Arginine Vasopressin, BD: Bipolar Disorder, CRH: Corticotropin-Releasing Hormone, DEX/CRH: Dexamethasone/Corticotropin-Releasing Hormone, FAM177A1: Family With Sequence Similarity 177 Member A1, FAM178B: Family With Sequence Similarity 178 Member B, GR: Glucocorticoid Receptor, GRAMD1B: GRAM Domain Containing 1B, GSK3B: Glycogen Synthase Kinase 3 Beta, HPA: Hypothalamic–Pituitary–Adrenal Axis, mRNA: Messenger RNA, MYO1H: Myosin 1H, NTRK2: Neurotrophic Tyrosine Kinase Receptor Type 2, OR52E2: Olfactory Receptor Family 52 Subfamily E Member 2, OR52J3: Olfactory Receptor Family 52 Subfamily J Member 3, 5-HTTLPR: Serotonin-Transporter-Linked Polymorphic Region

**Table 2:** Studies of the Effects of Lithium on BD-like and Other Psychiatric-like Behaviors in Animal Models

Animals and Methods	Behavioral Therapeutic Response	Main physiological findings	Reference
C57BL/6 Mice—treated for four weeks with control or lithium chow (2.4 g/kg of Li2CO3)	Increased neurons and glial cells in the dentate gyrus; higher astrocyte density	Lithium induces cellular proliferation in brain regions	[91]
Sprague–Dawley Rats—treated for five days with control or lithium (15 mEq/L in the drinking water)	Control of BD symptoms	Lithium and inositol combination attenuates adrenaline and dopamine release in the nucleus accumbens of rats with BD	[6]
Wister Rats—d-AMPH or saline for 14 days, and then, between days 8 and 14, rats were treated with lithium	Reversed and prevented hyperlocomotion induced by d-AMPH	No alterations in mitochondrial respiratory chain complexes I, II, III, and IV	[109]
Male Fisher CDF (F-344) Rats—treated with lithium for six weeks were injected i.p. 25 mg/kg NMDA daily for the last 21 days of lithium treatment	Lithium treatment mitigates NMDA-induced brain alterations, potentially enhancing its neuroprotective efficacy in conditions like BD	Chronic NMDA exposure induces mitochondrial dysfunction, lipid peroxidation, and altered complex I and III activity in the brain, highlighting mechanisms of glutamate-mediated excitotoxicity relevant to neurological disorders	[59]
Wistar Rats—Aβ1–42 oligomers-induced animal model of dementia, and oral treatments with memantine (5 mg/kg), lithium (5 mg/kg), or both drugs in combination for 17 days	Lithium monotherapy, in combination with memantine, improved spatial memory and reduced neuroinflammation	Reduced IL-4 and IL-1b levels, decreased TNF-a levels	[20]
Wistar Rats—LPS/CMS animal model and treatment with lithium (100 mg/kg/day) for four weeks	Improvement of depressive and cognitive deficits	Reduction of GSK-3 expression, tau hyperphosphorylation, and prevention of neuroinflammation	[35]
Sprague–Dawley Rats—ICSS to assess the effects of acute and chronic administration of lithium and aripiprazole (1 mg/kg each) for 21 days	Induction of tolerance to the anhedonic effect	Impact on neuroplasticity, induction of dendritic and synaptic growth	[75]

BD: Bipolar Disorder, CMS: Chronic Mild Stress, d-AMPH: d-Amphetamine, GSK-3: Glycogen Synthase Kinase-3, IL-4 and IL-1b: Interleukin-4 and Interleukin-1 beta, LPS: Lipopolysaccharide, NMDA: N-methyl-D-aspartate, TLR4: Toll-like Receptor 4, TNF-a: Tumor Necrosis Factor-alpha, Wnt/ß: Wnt/ß-catenin pathway

**Table 3:** Studies of the Effects of Lithium on BD-like and Other Psychiatric-like Behaviors in In Vitro Models

Cells	Experimental Protocol	Cellular Response	Analyzed Physiological Mechanisms	Reference
SK-N-SH and HEK-293 cells	Investigation of HTR1B haplotypes on gene expression	Influence on mental and behavioral disorders	Regulation of HTR1B gene expression by various haplotypes and regulatory regions	[119]
Rat hippocampal pyramidal neurons	Study on acute effects of high-dose lithium treatment (30–150 mM)	Inhibition of glutamatergic and GABAergic transmissions	Preferential action on presynaptic terminals; differential inhibition of eEPSCs and eIPSCs	[113]
Immortalized human microglia cells	Effect of lithium on tryptophan breakdown via the kynurenine pathway	1. Inhibition of tryptophan catabolism via the kynurenine pathway 2. Reduced activity of IDO1	2. Lithium affects inflammation-induced tryptophan catabolism 2. Lithium acts anti-inflammatory by reducing IDO1 activity	[45]
Rat primary astrocyte, neuronal, and mixed neuro-astrocyte cultures	In vitro study to analyze the exposure to lithium (1 mM) or vehicle	Increased BDNF regulation	Lithium prevents cellular degeneration by regulating BDNF	[36]
SH-SY5Y cells	Chronic lithium treatment (1.2 mM)	Improved mitochondrial function, membrane potential, and oxidation; protection against Meth-induced mitochondrial toxicity	Prevention of Meth-induced reduction in mitochondrial cytochrome c, Bcl-2/Bax ratio, and mitochondrial COX activity	[7]
Neuronal-derived cells (SH-SY5Y)	Lithium was added for the last 24/48 h of the exposure to rotenone for 72/96 h, respectively	Normalization of mitochondrial respiration and cell viability post-rotenone exposure	Implication of lithium in autophagy and mitochondrial function regulation, potential synergy with other autophagy enhancers	[28]
Human neuroblastoma cells (SH-SY5Y)	Assessment of protein oxidation with a lithium dose of 0.75 mM	Prevention of protein oxidation and nitration; maintenance of mitochondrial complex I activity and cell viability	Involvement of glutathione in lithium's antioxidant effects	[80]
Fibroblast cell lines	Treatment with a lithium dose of 20 mM for 48 h	Preservation of cell viability and mitochondrial respiratory capacity; synergistic effects with other autophagy and translation inhibitors	Role of autophagy and cytosolic translation in mitochondrial health	[87]
SH-SY5Y human neuroblastoma cells	Monotherapy—lithium dose of 0.5 mM	Promotion of neuritogenesis	Increased cell density, neurite growth, and branching, organization of microtubules and neurofilaments	[81]
BV-2 microglia	1 μg/ml of LPS and pretreatment with 10 mM of lithium	Improved cognitive performance	TLR4 inhibition, reduction of systemic and hippocampal inflammation	[66]

BD: Bipolar Disorder, BDNF: Brain-derived neurotrophic factor, eEPSC: Excitatory Postsynaptic Current, eIPSC: Inhibitory Postsynaptic Current, GSK3: Glycogen Synthase Kinase 3, HEK-293: Human Embryonic Kidney 293 cells, HTR1B: Serotonin receptor 1B gene, IDO1: Indoleamine 2,3-dioxygenase 1, LPS: Lipopolysaccharide, Meth: Methamphetamine, SK-N-SH and SH-SY5Y: cell lines

## Lithium, Toxicity and Side Effects

The most common adverse effects caused by the use of lithium are acne, increased appetite and weight, edema, loose stools, metallic taste, nausea, polydipsia, polyuria, increased creatinine levels, and fine tremors, as well as decreased memory and other cognitive functions. Less common symptoms include alterations in the cardiovascular, renal, and hepatic systems, as well as hair loss and alopecia, anorexia, ataxia, increased intracranial pressure, goiter, cavities, headache, seizures, diarrhea, dystonia, fatigue, muscle weakness, vomiting, dizziness, and blurred vision. Some uncommon symptoms are hyperparathyroidism, hypernatremia, and sexual dysfunction. Adverse effects may even contribute to the discontinuation of medication [43, 83].

In healthy individuals, the use of lithium at a dose between 1050 and 1950 mg twice a day for three weeks impairs the performance of short-term memory tasks compared with the results obtained in the same tests two weeks after discontinuing the use of the medication. In addition, learning a task is negatively influenced by the use of lithium. In the analysis of long-term memory, subjects who used lithium remembered fewer words than the placebo group. In short, the drug does not explicitly interfere with the memory and attention of healthy individuals, but it does influence learning [104].

Another research carried out with healthy individuals identified that the use of lithium for six months induces tremors in the hands (more frequently in individuals over 60 years of age) and thirst/polyuria, unrelated to the serum levels of the drug. The mechanism by which lithium-induced tremors occur in healthy subjects is unclear, so further research is needed. Thirst is a symptom of lithium-induced polyuria from inhibiting the kidneys’ response to antidiuretic hormone [11].

The chronic effects of lithium on kidney function were evaluated in a controlled clinical trial over an average of 11.5 years per person, totaling 61 cases and 53 controls. Lithium moderately decreased the glomerular filtration rate compared to control subjects. The percentage of people with grade 3 chronic kidney disease was 34.4% in the lithium-treated group compared to the control group, which totaled 13.1% [108].

Intoxication involves several severe symptoms that can progress to coma and death. Initially, the symptoms involve dysarthria, ataxia, and gross tremor, which may go to nausea, vomiting, abdominal pain, dry mouth, profuse diarrhea, lethargy or excitement, vertigo, alteration in the level of consciousness, cardiac arrhythmias, muscle fasciculations, hyperreflexia, delirium, nystagmus, convulsions, oliguria, and anuria [77].

Risk factors for lithium intoxication include overdose, renal impairment, low-sodium diet, drug interactions, and dehydration. If symptoms appear, it is necessary to stop using the drug immediately and call for medical help. It is also essential that the patient drinks plenty of water, considering that the kidneys excrete lithium. For this same reason, hemodialysis is one alternative to removing toxic medication levels from circulation [77].

## Discussions and Conclusions

Lithium remains a cornerstone in the treatment of BD, and its complex therapeutic profile continues to be refined through advances in molecular genetics, neurobiology, and translational research. However, understanding the full extent of lithium’s efficacy is complicated by key gaps, notably: (1) the heterogeneity of patient response, which limits its application to a subset of individuals; (2) the precise convergence of its multiple molecular targets (e.g., GSK-3, inositol pathway, mitochondrial function) on a unified mechanism of action; and (3) defining the therapeutic index that separates its neuroprotective benefits from its potential neurotoxic effects. The variability in clinical response among patients with BD has been increasingly attributed to genetic factors, with specific polymorphisms in genes such as GSK3B, NTRK2, ADCY1, FAM177A1, GRAMD1B, and ZNF804A showing strong associations with treatment outcomes. These genetic influences highlight the importance of intracellular signaling pathways, particularly those involving GSK-3 inhibition, which has emerged as a central mechanism underlying lithium’s mood-stabilizing properties. The polygenic load for major depression also appears to modulate treatment efficacy, suggesting a broader genomic context must be considered in understanding lithium response variability.

Neuroimaging and postmortem studies have supported lithium’s ability to increase grey matter volume and enhance neuroplasticity, indicating robust neurotrophic effects. Its influence on key neurotransmitter systems, including serotonergic, dopaminergic, and glutamatergic circuits, underscores its impact on mood regulation. Notably, lithium modulates inositol signaling, reduces intracellular inositol levels, and sensitizes neurons to serotonin, which may explain its efficacy in treatment-resistant depression and its augmentation of antidepressant effects. The dose-dependent, bimodal impact of lithium on hippocampal glutamate concentrations further highlights the delicate balance required to achieve optimal plasma levels, thereby maximizing efficacy while minimizing neurochemical disruption.

Animal model studies reinforce these findings, demonstrating lithium’s capacity to reverse BD-like behaviors, normalize stress responses, and reduce hyperlocomotion. Lithium promotes cellular proliferation in the dentate gyrus, increases astrocyte density, and exerts protective effects against excitotoxicity and neuroinflammation. These effects are mediated through pathways involving GSK-3 inhibition, Wnt/β-catenin activation, and reduced pro-inflammatory cytokine expression. In models of chronic stress, LPS exposure, or amyloid-induced neurotoxicity, lithium improves behavior, enhances memory, and mitigates cellular damage, suggesting applications that may extend to mood, cognitive, and neurodegenerative disorders.

In vitro studies provide further mechanistic insight, showing that lithium enhances mitochondrial function, supports oxidative balance, and prevents apoptosis through mechanisms involving cytochrome c regulation, Bcl-2 modulation, and preservation of complex I activity. Lithium influences gene expression patterns, including BDNF upregulation, and prevents protein nitration and oxidation. It reduces microglial activation, modulates the kynurenine pathway by inhibiting IDO1, and suppresses inflammatory responses via TLR4 inhibition. These cellular actions align with the observed behavioral and cognitive benefits in preclinical models, emphasizing lithium’s role in maintaining neuroimmune balance and mitochondrial integrity.

While lithium’s therapeutic efficacy is well established, its clinical utility is constrained by a narrow therapeutic index and the risk of adverse effects, including renal and thyroid dysfunction. The demonstrated variability in genetic and physiological response supports the need for personalized treatment strategies guided by pharmacogenomic profiling. However, a critical challenge remains that single-gene markers currently lack the predictive power for routine clinical use. Additionally, lithium’s capacity to regulate autophagy, stabilize synaptic architecture, and modulate neuroendocrine function positions it as a promising agent beyond psychiatry, with potential relevance to neurodegenerative and neuroinflammatory conditions.

In conclusion, lithium exhibits a wide range of therapeutic actions, including genetic regulation, neurotransmission, neurotrophic support, mitochondrial function, and immune modulation. Continued investigation into its mechanisms of action will be crucial for optimizing its use, improving safety profiles, and expanding its applications. Advances in precision medicine and biomarker discovery hold promise for enhancing lithium’s clinical impact and tailoring treatment to individual biological profiles, ensuring that this historically significant drug remains at the forefront of neuropsychiatric therapeutics.

## Data Availability

No datasets were generated or analysed during the current study.
